# SPIN90, an adaptor protein, alters the proximity between Rab5 and Gapex5 and facilitates Rab5 activation during EGF endocytosis

**DOI:** 10.1038/s12276-019-0284-5

**Published:** 2019-07-29

**Authors:** Hwan Kim, Hyejin Oh, Young Soo Oh, Jeomil Bae, Nan Hyung Hong, Su Jung Park, Suyeon Ahn, Miriam Lee, Sangmyung Rhee, Sung Haeng Lee, Youngsoo Jun, Sung Hyun Kim, Yun Hyun Huh, Woo Keun Song

**Affiliations:** 1 Cell Logistics and Silver Health Research Center, School of Life Sciences, Gwangju Institute of Science and Technology, Gwangju, 61005 Republic of Korea; 2 Center for Vascular Research, Institute for Basic Science, Daejeon, 34141 Republic of Korea; 3 Division of Basic Sciences, Fred Hutchinson Cancer Research Center, Seattle, WA 98109 USA; 4 Department of Life Science, Chung-Ang University, Seoul, 06974 Republic of Korea; 5 Department of Cellular and Molecular Medicine, Chosun University School of Medicine, Gwangju, 61452 Republic of Korea; 6 Department of Physiology, School of Medicine, Kyung Hee University, Seoul, 02447 Republic of Korea

**Keywords:** Endocytosis, Small GTPases, Growth factor signalling

## Abstract

During ligand-mediated receptor endocytosis, the small GTPase Rab5 functions in vesicle fusion and trafficking. Rab5 activation is known to require interactions with its guanine nucleotide-exchange factors (GEFs); however, the mechanism regulating Rab5 interactions with GEFs remains unclear. Here, we show that the SH3-adapter protein SPIN90 participates in the activation of Rab5 through the recruitment of both Rab5 and its GEF, Gapex5, to endosomal membranes during epidermal growth factor (EGF)-mediated endocytosis. SPIN90 strongly interacts with the inactive Rab5/GDI2 complex through its C-terminus. In response to EGF signaling, extracellular signal-regulated kinase (ERK)-mediated phosphorylation of SPIN90 at Thr-242 enables SPIN90 to bind Gapex5 through its N-terminal SH3 domain. Gapex5 is a determinant of Rab5 membrane targeting, while SPIN90 mediates the interaction between Gapex5 and Rab5 in a phosphorylation-dependent manner. Collectively, our findings suggest that SPIN90, as an adaptor protein, simultaneously binds inactive Rab5 and Gapex5, thereby altering their spatial proximity and facilitating Rab5 activation.

## Intracellular transport: a new SPIN

A key protein required for incorporating substances into cells has been identified by researchers in South Korea. During this process, called endocytosis, materials are engulfed by the cell membrane, then packaged into vesicles by organelles called endosomes for transport around the cell. The small enzyme Rab5 is involved in fusing and transporting vesicles through interactions with a protein called Gapex5. It has been unclear how these interactions occur, but Woo Keun Song and Yun Hyun Huh at Gwangju Institute of Science and Technology and co-workers suspected a role for SPIN90, an ‘adapter protein’ that helps connect other molecules. They found that Rab5 and Gapex5 did not come close in mutant cells lacking SPIN90. In normal cells, SPIN90 interacted strongly with both Rab5 and Gapex5, recruiting them to endosomes where they operated together on vesicle formation.

## Introduction

Receptor-mediated endocytosis is involved in a variety of cellular processes, including the uptake of extracellular solutes^1^, regulation of ligand-mediated receptor signaling^2^, and trafficking of cell surface receptors, such as epidermal growth factor receptor (EGFR)^3^ and transferrin receptors^4^. Rab GTPases (Rabs), members of the Ras-like small (21–25 kDa) GTPase superfamily, play pivotal roles in vesicle trafficking. Rabs coordinate vesicle formation, motility, and tethering;^5^ define the routes of vesicles by marking functionally distinct endosomes, and organize dynamic trafficking and fusion at specific cellular locations.

Rabs function as molecular switches by alternating between two conformational states: a GTP-bound active form and a GDP-bound inactive form. The activity of Rabs is spatially and temporally controlled by both guanine nucleotide-exchange factors (GEFs), which accelerate GDP release and subsequent GTP replacement, and GTPase-activating proteins (GAPs), which promote GTP hydrolysis. The conformational transition of Rabs between GTP and GDP-bound states is coupled to their shuttling between the cytosol and membranes, which is mediated by GDP dissociation inhibitors (GDIs). Double prenylation near the C-terminus of Rabs enables these proteins to associate with membranes, whereas GDI interactions with switch loops near the GDP-binding site and C-terminal prenyl groups induce the dissociation of Rabs from the membranes of their fusion targets^6^. Indeed, 10–50% of Rabs are detected in the cytosol^7^. GDI binding prevents GDP release and spontaneous GDP/GTP exchange on Rabs; therefore, Rab/GDI complexes occur in the cytosol in their inactive state^8^. Because GDI binds prenyl Rabs with a very high affinity^9^, it has been proposed that GDI-dissociation factors (GDFs) must be required. For instance, Yip3 (homolog of human PRA-1) has been identified as a protein with GDF activity toward Rab9 distinct from that of GEFs^10^, and SidM/DrrA from *Legionella pneumophila* has been shown to act as both a GEF and a GDF for Rab1^11–13^. However, the existence of GDFs in mammals is a matter of controversy because few GDFs have actually been found. It was recently asserted that GDFs are not thermodynamically required for membrane targeting of Rabs; instead, it was proposed that GEFs have the potential to dissociate GDIs from Rab/GDI complexes^6^. Thus, the controversies surrounding the mechanisms of GDI release from GDI-Rab complexes and specific targeting of Rabs to their fusion membranes remain largely unresolved.

Among the Rabs, Rab5, a key regulator of early endosome dynamics, controls clathrin-mediated vesicle formation^14^, early endosome fusion^15^, early-to-late endosome maturation^16^, and motility along microtubules^17^. Rab5 participates in early endosome fusion through early endosome antigen-1 (EEA1)^15^, Rabenosyn-5^17^, and phosphoinositol-3-phosphate^18^. Inhibition of Rab5 by treatment with an anti-Rab5 antibody blocks endosome fusion in vitro;^19^ overexpression of Rab5 induces endosome enlargement^5^, and a constitutively active mutant of Ras (Ras G12V) enhances Rab5 activity in association with the formation of markedly enlarged endosomes^20^. The overexpression of constitutively active mutant Rab5 (Rab5-Q79L) leads to increased homotypic fusion of early endosomes and eventual formation of oversized endosomes, whereas the expression of constitutively inactive mutant Rab5 (Rab5 S34N) induces the accumulation of small endosomes and inhibits the uptake of endocytic cargo into cells^21^. In mammals, Rab5 is regulated by multiple GEFs, including Rabex5^22^, Rin1^23^, Alsin^24^, and Gapex5^25^, all of which contain a conserved VPS9 domain, which is necessary and sufficient to support normal Rab5 GDP/GTP exchange^26^.

In a previous study, SPIN90 (SH3 protein interacting with Nck, 90kDa, also known as NCKIPSD) was suggested to be involved in endosome trafficking during epidermal growth factor (EGF)-mediated endocytosis of its cognate receptor (EGFR). SPIN90 knockdown (KD) was found to cause a delay in EGFR endocytosis, resulting in an increase in the level of surface EGFR, sustained activation of extracellular signal-regulated kinase-1/2 (ERK1/2), and enhanced cell proliferation^27^. Moreover, SPIN90 is involved in regulating actin dynamics and vesicle trafficking. In COS-7 cells, the proline-rich domain (PRD) of SPIN90 associates with syndapin I, which is required for clathrin-coated vesicle formation;^28,29^ the SH3 domain of SPIN90 binds dynamin I to catalyze the budding of vesicles from the plasma membrane in neuronal cells^30^. However, the molecular mechanisms by which SPIN90 functions in endosomal trafficking have not been elucidated. In the current study, we show that the C-terminus of SPIN90 interacts with the inactive Rab5a/GDI2 complex, and ERK-mediated phosphorylation of SPIN90 Thr-242 facilitates the interaction with Gapex5 (through its SH3 domain) on target membranes; therefore, the structural proximity of Gapex5 and Rab5 ultimately leads to Rab5 activation.

## Materials and methods

### Plasmids and primers

cDNA encoding full-length wild-type human SPIN90 was inserted into the pEGFP-c1 or pcDNA 3.0-HA vector (Clontech, Fremont, CA, USA). Thr-181 and/or Thr-242 alanine substitution mutants of SPIN90 (T181A, T242A, and TDA) were generated using a QuickChange site-directed mutagenesis kit (Stratagene San Diego, CA, USA). pKH3-Gapex5 constructs were kindly provided by Dr. M. Matsuda (Kyoto University, Japan) and Dr. A. R. Satiel (University of Michigan, USA), and pECFP-c1-Rab5a and pGEM-Rabaptin5 constructs were kindly provided by Dr. M. Zerial (Max Plank Institute, Germany). mCherry-FRB-FRB-ActA and FKBP-FKBP-eGFP vectors were provided by Dr. A. Itzen (Technische Universität München, Germany). Q79L and S34N mutants of Rab5a were cloned into pECFP vectors. Gapex5-Full, Gapex5-ΔVPS9, and Gapex5-VPS9 were subcloned into the pKH3 vector, and R5BD (Rabaptin5, 789-862) was subcloned into the pGEX-4T-1 vector.

### Cell culture and transfection

SPIN90 KD were generated by infecting HeLa cells with small interfering RNA (siRNA) against SPIN90 using the Mission RNAi system (Sigma, St. Louis, MO, USA), as described previously^27^. HEK293T cells were transfected with various constructs for binding assays and GST pulldown assays. COS-7 cells were used for immunofluorescence staining and live-cell imaging. Lipofectamine 3000 or LTX supplemented with plus reagent was used for transfections. Transfected cells were cultured for 24 h, followed by serum starvation for 16 h. HeLa-control, HeLa-SPIN90-KD, HEK293T, and COS-7 cells were authenticated by morphological analysis and gene expression profiling in 2015 and were tested for mycoplasma contamination using the MycoAlert mycoplasma detection kit (Lonza, Rockland, ME, USA) in 2017. Cell lines were usually used from passages 3 to 8.

### Immunofluorescence staining

For immunofluorescence staining, cells on 18-mm coverslips were fixed with 4% paraformaldehyde in phosphate-buffered saline containing 0.1 mM CaCl_2_ and 1.0 mM MgCl_2_ and were permeabilized by incubating with 0.5% Triton X-100 for 5 min. After blocking nonspecific binding with bovine serum albumin, cells were stained with the indicated antibodies for 30 min at 37 °C. Images were acquired with an FV1000 confocal microscope (Olympus, Tokyo, Japan) and analyzed using MetaMorph software.

### Pulse-chase assay for EGF trafficking

After being serum-starved for 16 h, SPIN90-KD and control HeLa cells were treated with Texas Red-EGF (40 ng/ml) for 5 min (pulse). Cells were washed with deferoxamine mesylate-containing serum-free media and incubated for an additional 45 min in serum-free media (chase). EGF localization with EEA1-positive early endosomes or LAMP1 (lysosomal-associated membrane protein 1)-positive late endosomes/lysosomes in confocal microscope images was quantified using MetaMorph software. The percentage of colocalized signals within single cells was integrated using the colocalization measurement function of MetaMorph software, and differences were statistically evaluated using a regression analysis.

### GST pulldown and Rab5 activation assays

GST-fused recombinant proteins were immobilized on glutathione-Sepharose beads (Incospharm, Daejeon, Korea) in buffer A (20 mM Tris-HCl pH 8.0, 1 mM EGTA, 150 mM NaCl, 0.5% Triton X-100, 0.5% sodium deoxycholate, 1 mM PMSF). Cells were lysed in binding buffer B (25 mM HEPES, 100 mM NaCl, 5 mM MgCl_2_, 1% NP-40, 10% glycerol, 1 mM PMSF), and lysates were incubated with bead-immobilized recombinant proteins. After incubation for 4 h at 4 °C, beads were washed four times with binding buffer B and analyzed by immunoblotting. The GTP-bound form of the Rab5-binding domain (R5BD) of Rabaptin5 was used for Rab5 activation assays.

### Rab5a/GDI2 complex and size-exclusion gel-filtration chromatography

Prenylated Rab5a was purified from the BJ5459 yeast line. Wild-type Rab5a was inserted into the pYES2 NT/C vector, and GST-GDI2 was subcloned into the pYES3-CT vector. Transfected yeast cells were cultured on URA-TRP/-CSM plates to select yeast clones that expressed both His-Rab5a and GST-GDI2. Lysates were purified using glutathione-Sepharose beads followed by Ni-NTA bead (Qiagen, Hilden, Germany) purification. Rab5a/GDI2 was purified by size-exclusion fast performance liquid chromatography (FPLC), and eluents were concentrated using a 10-kDa Vivaspin column (Sartorius, Göttingen, Germany). MBP-tagged, full-length SPIN90 and ΔCC proteins were transduced into *Escherichia coli* BL-21 (DE3) RIL cells, and their expression was induced by incubation with 0.5 mM isopropyl β-D-1-thiogalactopyranoside for 16 h at 18 °C. SPIN90 proteins were eluted with 200 mM imidazole (pH 8.0) from amylose beads (NEBm Ipswich, MA, USA) and subjected to size-exclusion FPLC. Eluents were concentrated with a 50-kDa Vivaspin and used for western blotting. Reaction mixtures were loaded directly onto a 150-ml Pharmacia S200 gel-filtration column and run in buffer C (64 mM HEPES pH 8.0, 100 mM NaCl, 8 mM MgCl_2_, 2 mM EDTA, and 0.2 mM DTT) with no added detergent.

### Immunoprecipitation

Immunoprecipitation was performed as previously described^14^. Briefly, cells were cultured for 24 h and then serum-starved by incubating in serum-free DMEM for 12–16 h. After treatment with EGF (40 ng/ml) for the indicated times, cells were lysed in immunoprecipitation lysis buffer (20 mM Tris-HCl pH 8.0, 150 mM NaCl, 1% Triton X-100, 10 mM sodium pyrophosphate), freshly supplemented with 5 mM sodium orthovanadate, 50 mM sodium fluoride, and a proteinase inhibitor cocktail (Roche Life Science, Mannheim, Germany). Cell lysates were incubated with specific antibodies overnight at 4 °C and then incubated for 3 h with protein A/G-Sepharose beads (GE Healthcare, Uppsala, Sweden).

### Duolink proximity-ligation assay

The endogenous interaction of Rab5 and Gapex5 was observed using a Duolink kit (Sigma, St. Louis, MO, USA), as described by the manufacturer. Briefly, cells were incubated with mouse anti-Rab5 and rabbit anti-Gapex5 antibodies and then incubated with proximity-ligation assay (PLA) probes consisting of two unique synthetic oligonucleotides conjugated to secondary antibodies. Oligonucleotides bound to primary antibodies against interacting proteins separated by a distance of ≤40 nm of two adjacent molecules were hybridized and ligated; this amplified the fluorescence signals and acted as a reporter. The resulting fluorescent dots were detected with an FV1000 confocal microscope, and their intensities were measured and analyzed with MetaMorph software.

### Statistical analysis

All images were acquired using identical acquisition parameters, and all data are presented as the means ± standard error of the mean. Significance was determined using a paired or unpaired Student’s *t*-test. Values of *p < 0.05; **p < 0.01; and ***p < 0.001 were considered indicative of significance. Pearson’s correlation coefficient (PCC, r) for the colocalizations among mCherry-FRB-ActA, FKBP-eGFP-POI, and CFP-X was calculated via Fluoview (4.0a) software (Olympus, Tokyo, Japan) and Huygens Professional 18.04 (SVI, Hilversum, VB, Netherland) was used for deconvolution.

## Results

### SPIN90 interacts with inactive Rab5 during ligand-mediated early endosome formation

**Fig. 1:**
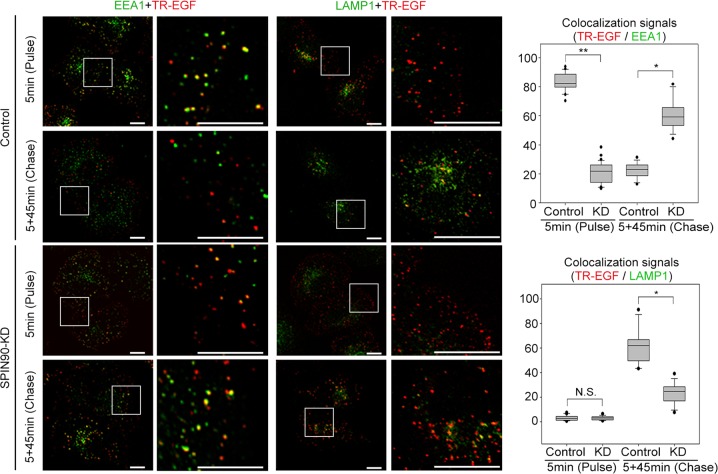
SPIN90-KD delays EGF-mediated early endosome formation. Pulse-chase assays were performed in SPIN90-KD and control HeLa cells using 40 ng/ml Texas Red (TR)-EGF. Early and late endosomes were labeled with anti-EEA1 and anti-LAMP1 antibodies, respectively. Scale bars, 20 μm; N.S. not significant; *p < 0.05 and **p < 0.01

To examine the function of SPIN90 throughout ligand-mediated endosome formation, we performed time-lapse observations of vesicle trafficking using pHrodo-conjugated EGF in HeLa cells stably depleted of SPIN90 (SPIN90*-*KD)^27^ generated by lentiviral-mediated delivery of a siRNA targeting SPIN90 and control cells expressing empty vector (Fig. S1a). The use of pHrodo-conjugated EGF enabled the tracking of specific internalized endosomes throughout EGF-stimulated endocytosis. EGF-mediated endocytosis was delayed in SPIN90*-*KD cells (Fig. S1b and Movie S1), as confirmed by pulse-chase analysis using Texas Red-conjugated EGF (TR-EGF; red) (Fig. 1). In SPIN90*-*KD cells, the number of EEA1 (an early endosome marker)-positive endosomes (green) containing TR-EGF (red; merged, yellow) was decreased at pulse (5 min) and was increased at chase (45 min after the removal of the TR-EGF-containing medium) compared with control cells (Fig. 1). Moreover, the number of LAMP1 (a late endosome marker)-positive endosomes (green) colocalized with TR-EGF (red; merged, yellow) was reduced by SPIN90 depletion at chase. These findings indicate that SPIN90 depletion attenuates early endosome formation and vesicle trafficking during ligand-mediated endocytosis, which is consistent with observations made in our previous work^27^.

**Fig. 2:**
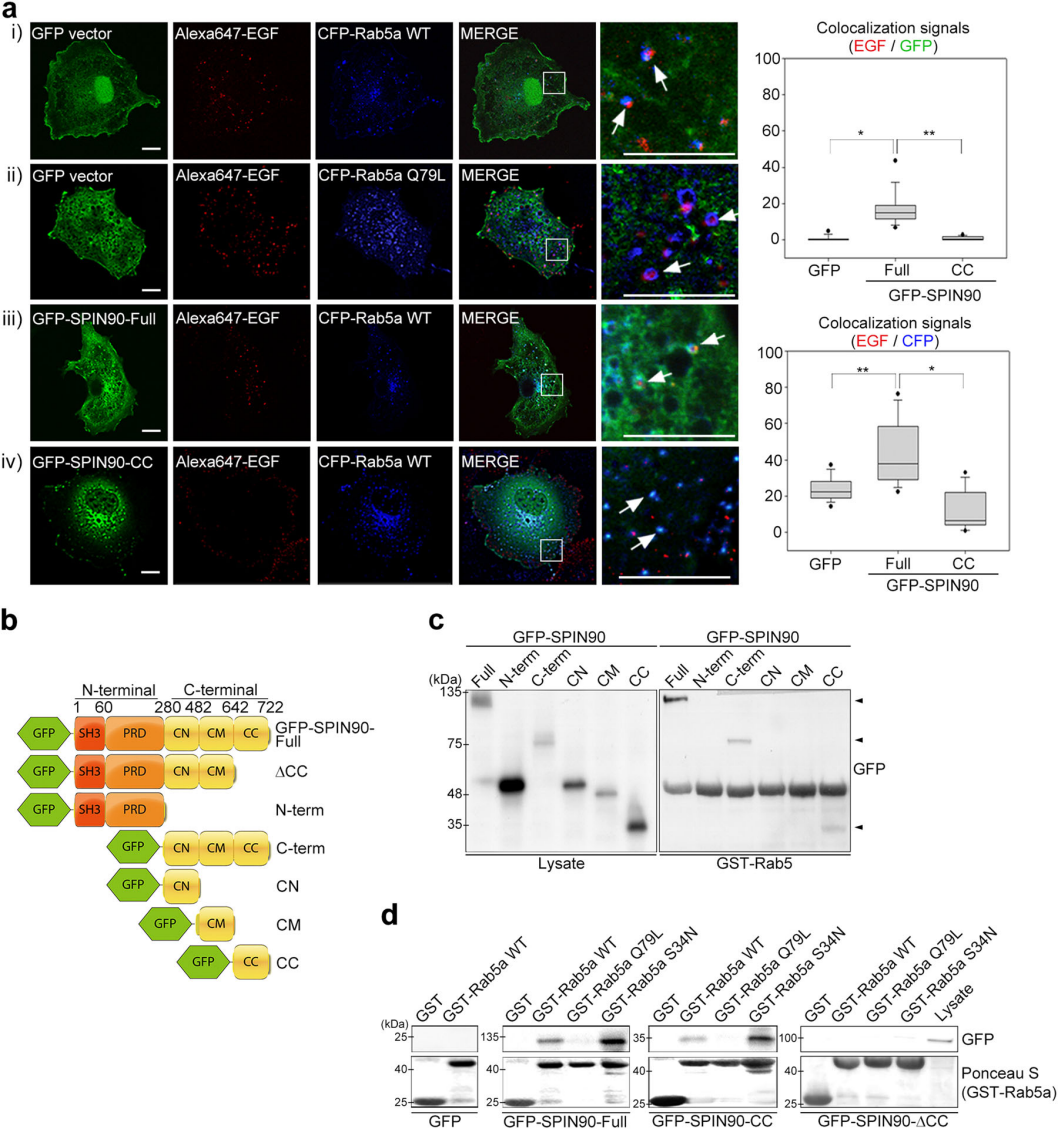
SPIN90 interacts with inactive Rab5 during EGF-mediated early endosome formation. **a** COS-7 cells transiently cotransfected with CFP-tagged Rab5 variants (WT or Q79L) and GFP-tagged SPIN90 variants (WT or CC) were serum-starved for 16 h and treated with 40 ng/ml Alexa 647-conjugated EGF for 10 min. Colocalization of EGF with SPIN90 or Rab5 was analyzed using MetaMorph software. Scale bars, 10 μm; *p < 0.05 and **p < 0.01. **b** Diagram of SPIN90 variant constructs. **c** Cells transfected with GFP-SPIN90-Full, GFP-SPIN90-N-term, GFP-SPIN90-C-term, GFP-SPIN90-CN, GFP-SPIN90-CM, or GFP-SPIN90-CC were used for the GST-Rab5 pulldown assay. **d** Cells were transfected with GFP, GFP-SPIN90-Full, GFP-SPIN90-CC, or GFP-SPIN90-ΔCC, and lysates were incubated with GST-Rab5 variants (GST-Rab5a-WT; GST-Rab5a-Q79L, active form; and the GST-Rab5a-S34N inactive form)

We next examined whether SPIN90 is involved in the function of Rab5, a key molecule in early endosome formation, during EGF-mediated endocytosis using truncated mutants of SPIN90 (Fig. 2a, b). Both SPIN90-Full and SPIN90-CC were dispersed in the cytoplasm in the absence of EGF (Fig. S2). After EGF treatment, most of the SPIN90-Full protein (GFP, green) was found to be colocalized with Rab5 (blue) in early endosomal membranes; in contrast, some SPIN90-CC protein was localized to endosomal membranes, while the majority remained in the cytosol (Fig. 2). Confocal microscopy images showed that wild-type Rab5a tagged with CFP (CFP-Rab5a-WT) was distributed in a punctate pattern (Fig. 2a, i), and CFP-Rab5a-Q79L (Rab5 GTP-bound mimetic) was localized in enlarged endosomes containing Alexa 647-EGF (Fig. 2a, ii), a result consistent with a previous study reporting that constitutive activation of Rab5 (Q79L) produces enlarged endosomes^3^. Interestingly, cotransfection of CFP-Rab5a-WT and full-length SPIN90 tagged with GFP (GFP-SPIN90-Full) resulted in the formation of enlarged Rab5-positive vesicles containing Alexa 647-EGF (Fig. 2a, iii), an effect similar to that produced by Rab5-Q79L. To further confirm the SPIN90 function in the enlargement of early endosomes, we used the distal end of the SPIN90 C-terminus (GFP-SPIN90-CC; Fig. 2b), which showed an inhibitory effect in EGFR endocytosis^27^. SPIN90-CC failed to induce the formation of enlarged endosomes or the colocalization of Alexa 647-EGF and Rab5 (Fig. 2a, iV). The quantification of these results showed that overexpression of GFP-SPIN90-Full enhanced the recruitment of SPIN90 as well as Rab5 to EGF-bound endosomes. In contrast, SPIN90-CC was not localized to EGF-bound endosomes (Fig. 2a, right panel), suggesting that SPIN90 might contribute to the formation of endosomes in association with Rab5. To verify the interaction between SPIN90 and Rab5, we carried out GST pulldown assays using SPIN90 and Rab5 variants. GST-Rab5a-WT strongly interacted with the C-terminus of SPIN90, especially the CC domain, but not with the N-terminus of SPIN90 (Fig. 2c). The activation status of Rab5 necessary for SPIN90 binding was determined using the GTP-bound mimetic Rab5a-Q79L and the GDP-bound mimetic Rab5a-S34N. These experiments revealed that GDP-bound Rab5 preferentially interacted with SPIN90 (Fig. 2d); SPIN90-CC also strongly interacted with inactive Rab5, but not SPIN90-ΔCC. Therefore, we conclude that SPIN90 interacts with inactive Rab5 and may function in Rab5 activation during EGF-mediated receptor endocytosis.

### SPIN90 directly binds to the Rab5a/GDI2 complex through its CC domain

**Fig. 3:**
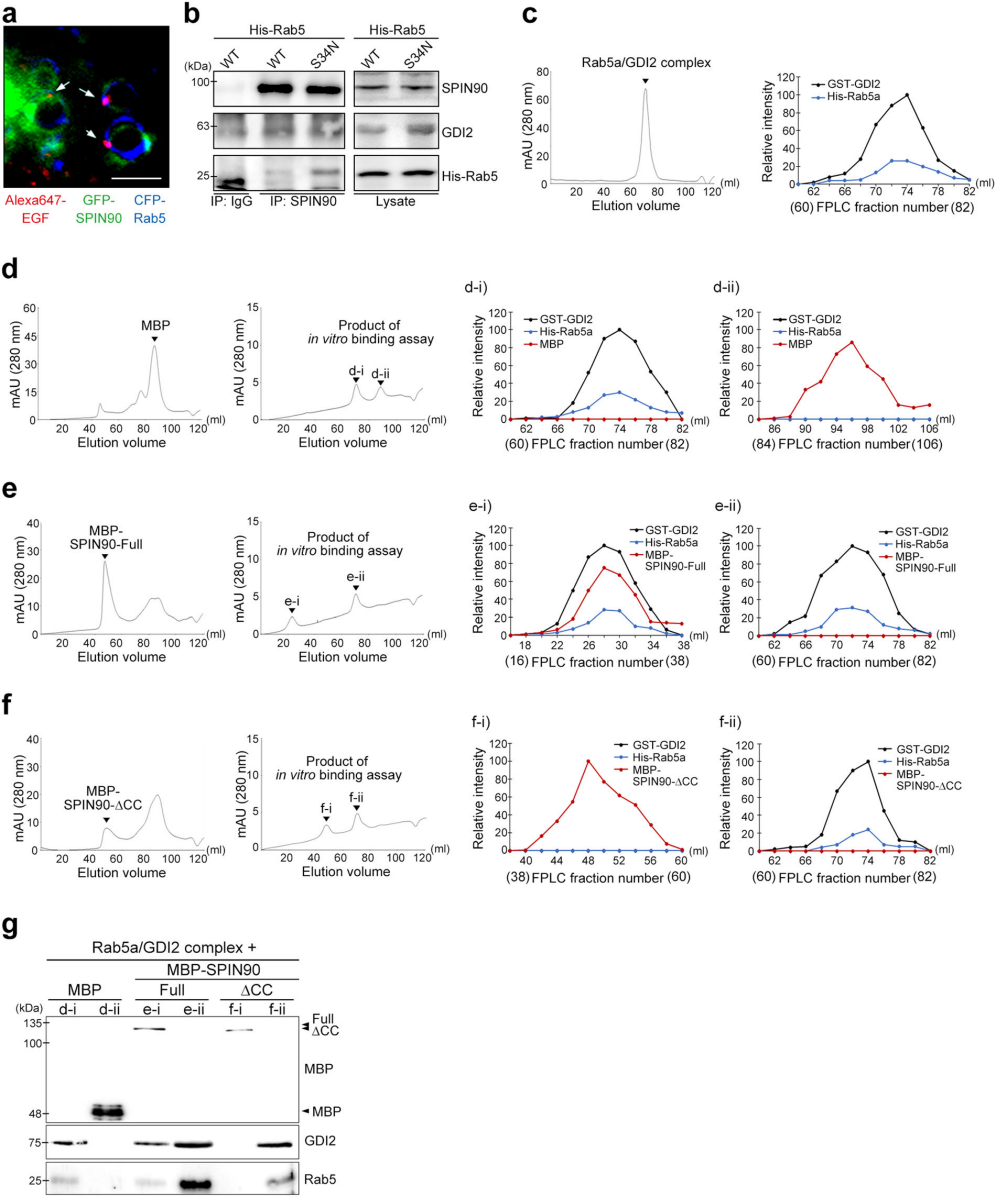
The SPIN90-CC domain interacts with the Rab5/GDI2 complex. **a** COS-7 cells transiently transfected with vectors encoding GFP-SPIN90 and CFP-Rab5 were serum-starved for 16 h. The cells were treated with 40 ng/ml Alexa 647-conjugated EGF, and time-lapse images were acquired every 10 s for 5 min. Representative captured images are displayed. Scale bar, 3 μm. **b** HEK293T cells were transfected with vectors encoding His-Rab5 (WT or S34N) and immunoprecipitated with an anti-SPIN90 antibody. **c**–**f** Binding status was analyzed by size-exclusion gel-filtration chromatography. The Rab5/GDI2 complex (**c**) was mixed with the MBP-tag (**d**), MBP-SPIN90-full (**e**), or MBP-SPIN90-ΔCC (**f**) and loaded onto a size-exclusion gel-filtration column. **g** The eluents corresponding to peaks d-(i, ii), e-(i, ii), and f-(i, ii) were analyzed by western blotting using anti-MBP, anti-GDI2, or anti-Rab5 antibodies

To trace the interaction between Rab5 and SPIN90, we performed live imaging analysis. Sequential captures of GFP-SPIN90 and CFP-Rab5a through confocal microscopy showed colocalization of SPIN90 and Rab5 on dynamically moving EGF-containing endosomes (Fig. 3a), indicating that SPIN90 might play a role in Rab5 activation and early endosome formation. The targeting of Rab5 to endosomal membranes (where GEFs are associated) and subsequent activation of Rab5 is regulated by the dissociation of GDI2 from the Rab5/GDI2 complex^6^. To verify the interaction between inactive Rab5/GDI2 and SPIN90, we used an anti-SPIN90 antibody to conduct immunoprecipitation assays on lysates of cells transfected with vectors encoding His-Rab5a (WT or S34N). As shown in Fig. 3b, SPIN90 precipitated well with inactive Rab5a (S34N)/GDI2 but showed less precipitation with WT Rab5a. Thus, we purified the Rab5a/GDI2 complex using His-Rab5a and GST-GDI2 derived from yeasts. The size-exclusion chromatography results showed one peak (fractions 60–82) corresponding to the Rab5a/GDI2 complex (Fig. 3c, left panel). Quantification of band intensities in western blots of fractions 60–82 verified the complex of GST-GDI2 and His-Rab5a (Fig. 3c, right panel). The binding ability of SPIN90 for the Rab5a/GDI2 complex was determined by performing size-exclusion chromatography using the Rab5a/GDI2 complex (collected eluents, fractions 60–82 in Fig. 3c) and recombinant MBP-SPIN90-Full (collected eluents, fractions 50–66 in Fig. 3e). After the reaction with Rab5a/GDI2, whereas MBP-tag used as a control did not associate with the Rab5a/GDI2 complex and was detected in fractions 84–106 (Fig. 3d; d–i, His-Rab5a/GST-GDI2; d-ii, MBP-tag), the peak corresponding to MBP-SPIN90-Full was shifted from fractions 50–66 to fractions 16–38 (Fig. 3e; e–i, MBP-SPIN90-Full/Rab5a/GDI2 complex; e-ii, His-Rab5a/GST-GDI2), indicating the association of SPIN90 with the Rab5a/GDI2 complex. Next, we sought to confirm the association of the SPIN90-CC domain and Rab5a/GDI2 complex, as shown in Fig. 2c, d. However, MBP-SPIN90-CC purified from bacteria was highly aggregated (Fig. S3) and could not be dissolved in several buffer compositions. Alternatively, we purified a CC-domain-deleted mutant of SPIN90 (MBP-SPIN90-ΔCC) and confirmed that the association between SPIN90-ΔCC and the Rab5a/GDI2 complex did not occur (Fig. 3f; f-i, MBP-SPIN90-ΔCC; f-ii, His-Rab5a/GST-GDI2), highlighting the importance of the SPIN90-CC domain for the interaction with the Rab5a/GDI2 complex. Western blotting of eluents corresponding to each peak (d-i, d-ii, e-i, e-ii, f-i and f-ii) also confirmed that the CC domain of SPIN90 is essential for its direct binding to the Rab5a/GDI2 complex (Fig. 3g). In addition, chromatography analysis revealed that the binding of SPIN90 to the Rab5a/GDI2 complex did not induce GDI2 release from the Rab5a/GDI2 complex (Fig. 3e). Collectively, these results indicate that SPIN90 directly binds to the Rab5a/GDI2 complex.

### ERK-mediated threonine phosphorylation of SPIN90 is required for its interaction with Gapex5

**Fig. 4:**
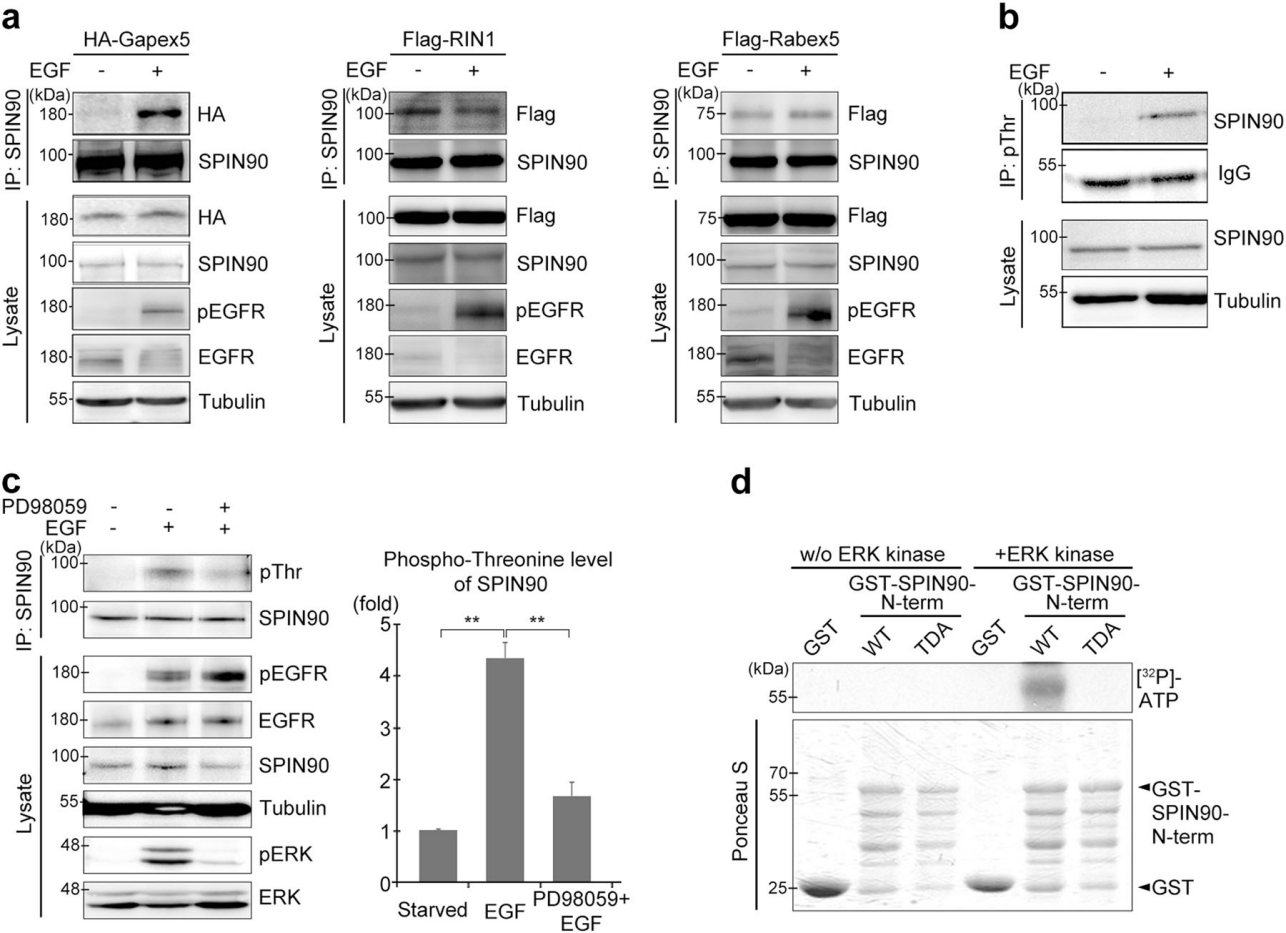
EGF-mediated ERK activation induces SPIN90 phosphorylation at Thr-242. **a** HEK293T cells transfected with HA-Gapex5, Flag-RIN1, or Flag-Rabex5 were treated with 40 ng/ml EGF for 10 min and immunoprecipitated with an anti-SPIN90 antibody. **b** Cells were incubated with or without EGF, and lysates were immunoprecipitated with an anti-pThr antibody. **c** Cells pretreated with 50 μM PD98059 for 30 min were stimulated with 40 ng/ml EGF for 10 min, and lysates were immunoprecipitated with an anti-SPIN90 antibody. **d** Cells transfected with SPIN90-WT or TDA (T181/242A) mutant were used for in vitro ERK kinase assays with [^32^P]-ATP

To investigate the involvement of SPIN90 in the activation of Rab5, we examined the interactions of SPIN90 with Rab5 GEFs, including Rin1, Rabex5, and Gapex5, because activation of Rab5 is mainly regulated by Rab5 GEFs. Our results revealed that Gapex5 strongly interacted with SPIN90 in response to EGF treatment, whereas the interactions of RIN1 and Rabex5 with SPIN90 were largely unaltered following EGF treatment (Fig. 4a). The strong interaction of Gapex5 with SPIN90 under EGF stimulation suggested that Gapex5 is a candidate GEF for Rab5 activation, whereas RIN1 or Rabex5 appear to function via other mechanisms. We next tested whether the interaction of SPIN90 with Gapex5 in response to EGF stimulation requires a posttranslational modification of SPIN90, such as phosphorylation. We found that EGF induced a marked increase in SPIN90 phosphorylation on threonine residues (Fig. 4b) but had no significant effect on tyrosine or serine phosphorylation of SPIN90, despite a trend toward increased phosphorylation of these residues (Fig. S4a, b). Two candidates for ERK-mediated threonine phosphorylation of SPIN90, Thr-181, and Thr-242, were predicted by the KinasePhos database (http://kinasephos.mbc.nctu.edu.tw/index.php). Consistent with this prediction, EGF-induced phosphorylation of SPIN90 threonine residues was significantly inhibited by blocking ERK activity with PD98059, a specific inhibitor of MEK1, the upstream kinase of ERK (Fig. 4c). In vitro kinase assays showed that, whereas wild-type SPIN90 was phosphorylated in response to EGF stimulation, the SPIN90 mutant (TDA, phosphorylation-deficient mutant), in which both Thr-181 and Thr-242 residues were replaced with alanine, was not (Fig. 4d).

**Fig. 5:**
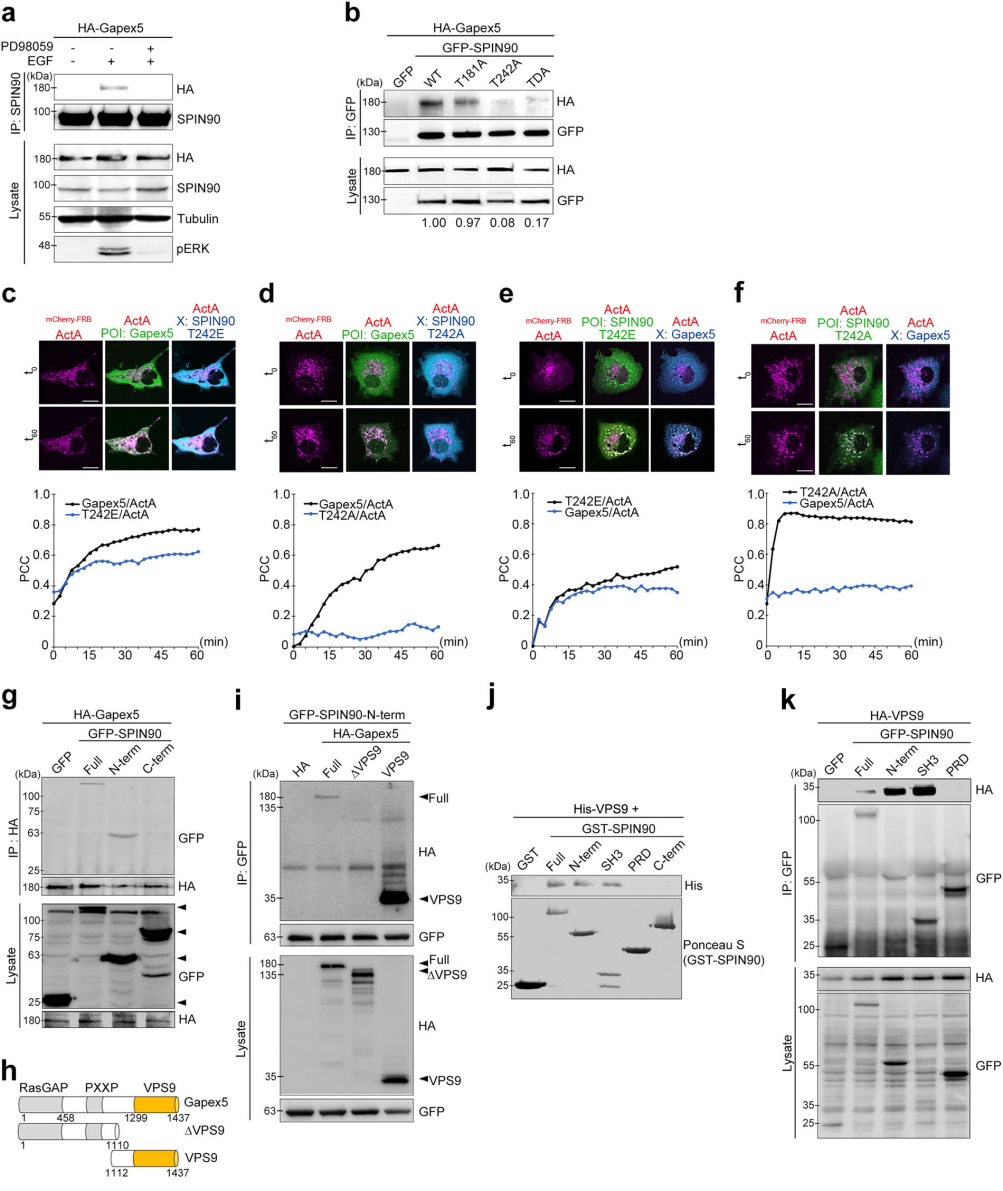
SPIN90 phosphorylation at Thr-242 is required for its interaction with Gapex5. **a** HEK293T cells transfected with HA-Gapex5 were treated with 50 μM PD98059 prior to EGF stimulation (40 ng/ml for 10 min). After immunoprecipitation with an anti-SPIN90 antibody, interactions with Gapex5 were analyzed by western blotting. **b** Cells were transfected with HA-Gapex5 and GFP-empty vector or GFP-SPIN90 variants (WT, T181A, T181E, or TDA). Lysates were immunoprecipitated with an anti-GFP antibody. **c**–**f** COS-7 cells were transfected with mitochondrial-localized mCherry-FRB-ActA, FKBP-eGFP-POI (protein of interest), and CFP-X (see Fig. S5a). Time-lapse images were acquired every 20 s for 60 min after the addition of A/C heterodimerizer; initial (t_0_) and final (t_60_) images are shown. Pearson’s correlation coefficient (PCC, r) for the colocalization between mCherry-FRB-ActA and FKBP-eGFP-POI or CFP-X was calculated. **c** POI-Gapex5, X-SPIN90-T242E; **d** POI-Gapex5, X-SPIN90-T242A; **e** POI-SPIN90-T242E, X-Gapex5; and **f** POI-SPIN90-T242A, X-Gapex5. Scale bars, 20 μm. **g** HEK293T cells transfected with HA-Gapex5 and GFP-SPIN90 variants were immunoprecipitated with an anti-HA antibody. **h** Diagram of truncated Gapex5 variants. **i** Cells transfected with HA-Gapex5 variants (Full, ΔVPS, or VPS) and GFP-SPIN90-N-term were immunoprecipitated with an anti-GFP antibody. **j** GST pulldown assays were performed using GST-SPIN90 variants (full, N-term, SH3, PRD, and C-term) and lysates obtained from cells expressing His-Gapex5-VPS. **k** Cells transfected with the HA-VPS domain of Gapex5 and GFP-SPIN90 variants (full, N-term, SH3, and PRD) were immunoprecipitated with an anti-GFP antibody

Next, we examined whether EGF-mediated phosphorylation of SPIN90 is required for the interaction with Gapex5. The EGF-mediated interaction of SPIN90 and Gapex5 was completely blocked when we inhibited ERK with PD98059 (Fig. 5a). A single point mutation that altered threonine to alanine at residue 242 (T242A) of SPIN90 efficiently inhibited the interaction with Gapex5, whereas the same change at Thr-181 did not (Fig. 5b). This finding indicates that phosphorylation at Thr-242 of SPIN90 is necessary for its interaction with Gapex5. This result was further supported by the kinase assay result, which showed that the T242A mutant of SPIN90 was not phosphorylated by ERK (Fig. S4c).

It has been shown that activated Rabs, including Rab5 and its effectors, act exclusively at the membrane and that GEFs are recruited to vesicle membranes before Rabs^31^. To determine whether SPIN90 is involved in the recruitment of Gapex5 and Rab5 to the target membrane, we utilized the rapamycin-induced protein heterodimerization system^31^, in which interactions between the FK506-binding protein domain (FKBP) and the FKBP-rapamycin-binding domain (FRB) are induced by the addition of the rapamycin analog A/C heterodimerizer (Fig. S5a). To apply this assay, we fused an FRB domain to both mCherry and the mitochondrial localization sequence from the *Listeria monocytogenes* protein Act A (mCherry-FRB-ActA), fused an FKBP domain to GFP as well as the POI (protein of interest; Gapex5, Rab5 or SPIN90; FKBP-GFP-POI), fused CFP to’X’ (CFP-X), and then tested whether CFP-X is recruited by FKBP-GFP-POI to mCherry-FRB-ActA on mitochondria (Fig. S5a). In control experiments, no mitochondrial translocation of CFP (empty vector) was observed in cells cotransfected with vectors encoding FKBP-GFP-Gapex5 (Fig. S5b) or FKBP-GFP-SPIN90 (Fig. S5c) and exposed to the heterodimerizer. In contrast, the addition of the A/C heterodimerizer caused the translocation of FKBP-GFP-Gapex5 to mitochondria, with concomitant translocation of CFP-T242E (Fig. 5c, t_60_) to mitochondria. Pearson’s correlation analysis (PCC) via Fluoview Software and Huygens Professional Software for deconvolution revealed real-time movement of Gapex5 and SPIN90 to mitochondria (Fig. 5c–f). As shown in Fig. 5c, d, phosphorylated SPIN90 (CFP-T242E) was more effectively translocated to mitochondria by FKBP-GFP-Gapex5 (Fig. 5c) than nonphosphorylated SPIN90 (CFP-T242A, Fig. 5d), suggesting that SPIN90 translocation to mitochondria by FKBP-GFP-Gapex5 is caused by the phosphorylation-dependent interaction of SPIN90 with Gapex5. Reciprocally, we examined the effects of FKBP-GFP-T242E or FKBP-GFP-T242A on the translocation of CFP-Gapex5. Gapex5 translocation to mitochondria was significantly induced by the mitochondrial localization of FKBP-GFP-T242E (Fig. 5e) but not by the localization of the nonphosphorylated form (FKBP-GFP-T242A) of SPIN90 (Fig. 5f). Interestingly, the colocalization of CFP-Gapex5 with FKBP-GFP-T242E was found even before A/C heterodimerizer treatment (t_0_), reflecting their strong interaction, which was not present with FKBP-GFP-T242A (Fig. S5d).

Next, we investigated which domains of Gapex5 and SPIN90 are involved in their interaction. First, we found that the N-terminus of SPIN90 was responsible for its interaction with Gapex5 (Fig. 5g), and then we performed coimmunoprecipitation assays using various deletion mutants of Gapex5 (Fig. 5h) and SPIN90 variants (Fig. 2b). These assays showed that the VPS9 domain of Gapex5 (residues 1112–1437) was essential for the interaction with the N-terminal SH3 domain of SPIN90 (Fig. 5i, k). GST pulldown assays performed using GST-SPIN90 variants also supported the idea that this interaction occurs via the VPS domain of Gapex5 and the SH3 domain of SPIN90 (Fig. 5j). From these data, we conclude that the N-terminal SH3 domain of SPIN90 interacts with the Rab5 GEF, Gapex5, when SPIN90 is phosphorylated at Thr-242 in the PRD domain (near the SH3 domain) via EGF signaling.

### SPIN90 mediates the Gapex5 interaction with Rab5 during EGFR endocytosis

**Fig. 6:**
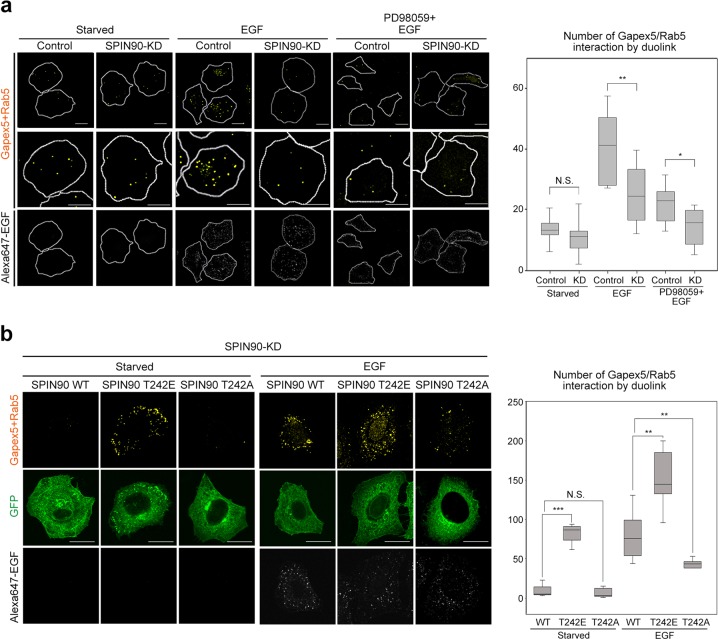
SPIN90 mediates the Gapex5 interaction with Rab5 during EGFR endocytosis. **a** SPIN90-KD and control HeLa cells were serum-starved for 16 h, pretreated with or without 50 μM PD98059 for 30 min, exposed to 40 ng/ml Alexa 647-EGF for 10 min and applied to a Duolink proximity assay. The cells were incubated first with anti-Gapex5 and anti-Rab5 and then with PLA probes. Fluorescent dots (yellow) in confocal microscopy images reflecting the proximity of Gapex5 and Rab5 were analyzed using MetaMorph software. Scale bars, 20 μm; N.S. not significant; *p < 0.05; **p < 0.01. **b** Cells transfected with SPIN90-WT or mutants (T242E or T242A) were treated with or without 40 ng/ml EGF for 10 min and applied to a Duolink proximity assay. Scale bars, 20 μm; N.S., not significant; **p < 0.01 and ***p < 0.001

Our demonstration that the CC domain of SPIN90 directly binds the Rab5a/GDI2 complex (Fig. 3) and that the SH3 domain of SPIN90 interacts with the VPS9 domain of Gapex5 in the context of EGF signaling (Fig. 5) suggests that the interaction with SPIN90 governs the spatial proximity of the inactive Rab5a/GDI2 complex and Gapex5. To test this hypothesis, we employed Duolink PLA technology, which is capable of detecting and visualizing endogenous protein–protein interactions in intact cells and identifying the cellular location in which the protein interaction occurs. In control HeLa cells, Alexa 647-EGF (white spots) treatment prominently increased the proximity between Rab5 and Gapex5 (Fig. 6a, yellow spots), and the effect was significantly blocked by the inhibition of ERK with PD98059 (Fig. 6a). In contrast, the close proximity of Rab5 and Gapex5 with EGF was not seen in SPIN90-KD cells (Fig. 6a). Then, we hypothesized that EGF-induced phosphorylation of SPIN90 at the Thr-242 residue is critical for the close proximity of Rab5 and Gapex5. As expected, ectopic expression of the phospho-mimetic T242E mutant increased the proximity of Rab5 and Gapex5 even under starvation condition; in contrast, the phospho-deficient T242A mutant did not exhibit increased proximity even in the presence of EGF (Fig. 6b). The slightly enhanced effects of EGF treatment on the proximity of Gapex5 and Rab5 in all WT-, T242E-, or T242A-overexpressing SPIN90-KD cells compared with control cells seemed to be caused by residual endogenous SPIN90 in SPIN90-KD cells (Fig. 1a). Taken together, these data suggest that EGF-mediated SPIN90 phosphorylation is crucial for increasing the Gapex5 and Rab5 proximity, which facilitates Rab5 activation.

### SPIN90 enhances Gapex5-mediated Rab5 activation

**Fig. 7:**
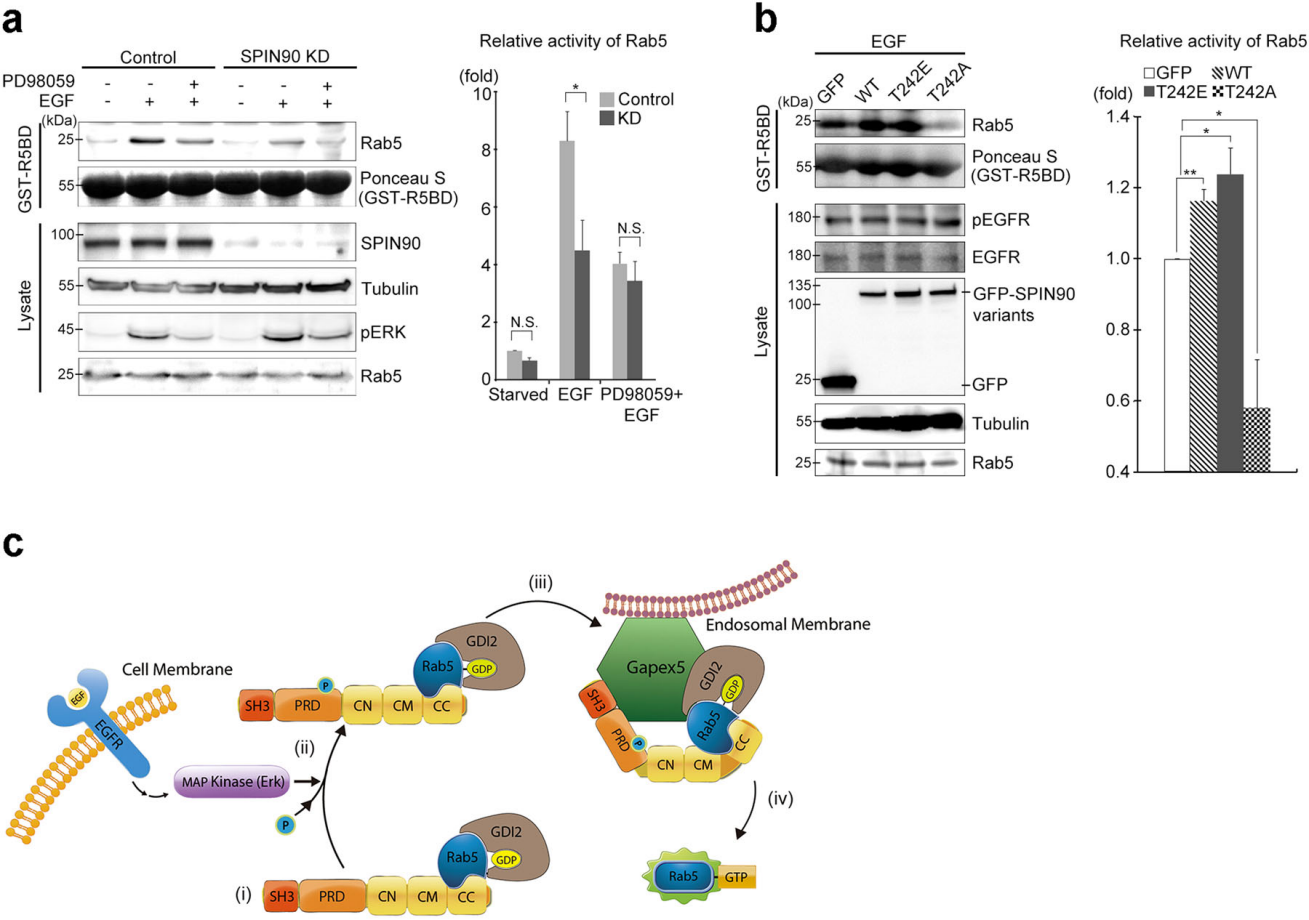
Rab5 activation requires an interaction between SPIN90 and Gapex5. **a** SPIN90-KD and control HeLa cells were pretreated with or without 50 μM PD98059 for 30 min and then treated with 40 ng/ml EGF for 10 min. Cell lysates were incubated with GST-R5BD and analyzed by western blotting using the indicated antibodies. **b** Cells transfected with GFP or GFP-SPIN90 variants (WT, T242E, or T242A) were incubated with EGF and used in GST-R5BD pulldown assays. **c** Proposed model for how SPIN90 regulates Rab5 activation via Gapex5. (i) The CC domain of SPIN90 interacts with the inactive Rab5/GDI2 complex. (ii) During the response to EGF, Thr-242 (located in the PRD) of SPIN90 is phosphorylated by ERK. (iii) This phosphorylation facilitates the interaction of the SPIN90/Rab5/GDI2 complex with Gapex5 on endosomal membranes. (iv) The structural proximity of Gapex5 and Rab5 on endosomal membranes enables the activation of Rab5 via Gapex5-mediated GTP-GDP exchange, which promotes the fusion and trafficking of early endosomes

To verify the importance of the interaction between SPIN90 and Gapex5 for Rab5 activation, we examined Rab5 activation using a recombinant Rab5-binding domain of Rabaptin5 (GST-R5BD; residues 789–862), which specifically interacts with active GTP-bound Rab5. We found that EGF-induced Rab5 activation was reduced in SPIN90-KD cells compared with the control cells, and abrogation of ERK activation by PD98059 pretreatment inhibited EGF-induced GTP loading of Rab5 in both control and SPIN90-KD cells (Fig. 7a). EGF stimulation induced an increase in Rab5 activity in cells overexpressing GFP-SPIN90-WT, but Rab5 activity was completely blocked in cells overexpressing GFP-SPIN90-T242A in the context of EGF signaling (Fig. 7b). These findings suggest that the CC domain of SPIN90 interacts with Rab5, that phosphorylation of SPIN90 Thr-242 facilitates the interaction with Gapex5 on target membranes, and that the structural proximity of Gapex5 and Rab5 ultimately leads to Rab5 activation (Fig. 7c).

## Discussion

Ligand-bound cell surface receptors are internalized into the cell, where they are recycled or degraded through transport to lysosomes, resulting in a dramatic decrease in cell surface receptors and downregulation of receptor signaling. In our previous study, we found that trafficking of EGFR-containing vesicles is delayed in SPIN90-KD cells, leading to an increase in surface EGFR levels and cell proliferation through enhanced ERK/cyclin D1 activity^27^. Under basal conditions, ligand-free EGFRs are usually recycled to the plasma membrane, whereas activation of EGFR by agonists provokes an interaction with signaling proteins and endocytic regulators. The association of EGFRs with endosomal components transduces activated receptor signals to downstream targets; thus, endosomal compartments act as intermediates in signaling between the plasma membrane and nucleus^32^. Because endocytosis is critical for the maintenance of cellular homeostasis, it is spatially and temporally controlled. Rab5, acting as a molecular switch, regulates endocytic trafficking and vesicle-endosome fusion in the early stage of ligand-mediated endocytosis^33^. Based on our previous study demonstrating that SPIN90 participates in the early stage of vesicle trafficking and vesicle-endosome fusion during EGFR endocytosis^27^, we investigated SPIN90 function in Rab5 activation during EGF-mediated endocytosis. We found that overexpression of SPIN90 induced the formation of enlarged endosomes, similar to those induced by Rab5-Q79L, and that the SPIN90-CC domain directly binds the Rab5a/GDI2 complex. During EGF-mediated endocytosis, phosphorylation of SPIN90 Thr-242 by ERK enables SPIN90 binding to Gapex5, which increases the proximity of Rab5 to its GEF, Gapex5, on target membranes.

In terms of the role of SPIN90 in Rab5 activation, we initially assumed that SPIN90 could possibly act as a GEF for Rab5. This function would presumably require the highly conserved VPS9 domain of Rab5 GEFs, which catalyzes the GDP/GTP exchange reaction in Rab5 family members^26^. However, we found no sequence homology between SPIN90 and the VPS9 domains of several known GEFs, including Rabex5, Rin1, and Gapex5 (data not shown), suggesting that SPIN90 itself does not possess GDP/GTP exchange activity. Thus, we next investigated the possibility that SPIN90 acts as a GDF. GDP-bound Rab5 generally binds to a GDI, which masks hydrophobic prenyl groups and the GDP-binding pocket to prevent uncontrolled GDP release. Completion of the Rab5 activation process requires the release of the GDI to allow GDP/GTP exchange by GEFs. To date, GDF function has been identified in two proteins: Yip3 in plants^10^ and SidM/DrrA in *L. pneumophila*^12,13^. However, the existence of GDFs is a matter of controversy because human GDFs have not yet been identified, and it has been suggested that the GEF activity of SidM/DrrA is sufficient to displace GDI and convert GDP to GTP^34^. Moreover, a relatively large amount of SidM (K_d_ for SidM and Rab1 binding ≈84 nM) is needed to overcome the affinity between Rab1 and its GDI (*K*_*d*_ ≈ 20 nM)^13^. In this study, size-exclusion assays using FPLC revealed a tripartite complex of SPIN90/GDI2-Rab5, indicating that SPIN90 is unlikely to possess GDF activity.

SPIN90 preferentially interacted with GDP-bound Rab5 through its CC domain; however, overexpression of the SPIN90-CC domain reduced the formation of EGF-bound/Rab5-positive vesicles (Fig. 2a, iV). This dominant-negative effect of SPIN90-CC may be explained by the strong interaction of overexpressed SPIN90-CC with inactive Rab5. These observations also suggest that certain proteins that interact with other domains of SPIN90 might be critical regulators of Rab5 activation. We thus assessed the interactions of SPIN90 with Rab5 GEFs. We found that the SH3 domain of SPIN90 interacts with the VPS9 domain of Gapex5 in a manner that depends on the phosphorylation status of SPIN90, implying a structural modification. We further found that phosphorylation of Thr-242 in the PRD near the SH3 domain of SPIN90 was mediated by ERK1/2 signaling and crucial for Gapex5 binding. It is likely that a conformational change in SPIN90 induced by phosphorylation results in the exposure of a Gapex5-binding region, thereby enabling SPIN90 to bind to Gapex5. The increased proximity of GDI2-Rab5 to Gapex5 resulting from its interaction with SPIN90 might facilitate GTP loading of Rab5 by Gapex5 and enable activated GTP-bound Rab5 to subsequently activate the Rab5 effector. A predominant aspect of the role of SPIN90 in Rab5 activation during EGFR endocytosis is its adaptor function, which facilitates its association with both the Rab5/GDI2 complex and its GEF, Gapex5. A previous report proposed a different mechanism for mediating interactions of Rab5 and its GEF, Rabex5, whereby immobilized Rab5 on the membrane recruits a soluble complex of Rabex5 and Rabaptin5, which attenuates the GTP hydrolysis of Rab5, resulting in the amplification of Rab5 activation^22^. According to this model, Rabex5 and Rabaptin5 are tightly bound, and the complex does not dissociate after Rab5 activation, whereas binding of SPIN90 and Gapex5 is dependent on the phosphorylation status of SPIN90, as determined by EGF-induced ERK signaling. Our current study provides novel insights into this process, demonstrating that SPIN90, acting as an adaptor that binds both Rab5 and its GEF, might be a signal-mediated, finely tuned regulator of Rab5 activation during EGFR endocytosis. Since Rab5 activation occurs on a membrane, it must be recruited to target vesicles^31^. In the current study, we investigated the contribution of SPIN90 to the targeting of Rab5 to membranes using the rapamycin-induced protein heterodimerization system. After acute, artificial targeting of Gapex5 to the mitochondrial membrane, phosphorylated SPIN90 was concomitantly redistributed to mitochondria. These results are consistent with previous reports that Rabex5 recruits Rab5^31^ or DrrA/SidM recruits Rab1 specifically to intracellular compartments^12^. The intrinsic localization properties of GEFs might determine the specific Rab membrane localization; similarly, Gapex5 might steer Rab5 to the endosomal membrane. In addition, SPIN90 mediates membrane targeting of inactive Rab5 depending on the phosphorylation status. During EGFR endocytosis, the ERK-mediated phosphorylation of SPIN90 Thr-242 drives SPIN90 toward Gapex5, allowing the C-terminal domain of SPIN90 to interact with Rab5, thus explaining the proximity between Gapex5 and Rab5.

A prominent aspect of this study is the revelation of the adaptor function that allows SPIN90 to simultaneously interact with both the GDI2-Rab5 complex and its GEF, Gapex5. Although it has been reported that Bem1 is capable of simultaneously binding cdc42, a member of the small GTPases, and its GEF, Cdc24, the function and regulatory mechanisms of the cdc42 family are quite different from those of Rabs^35^. Whereas Bem1 binds activated Cdc42 and Cdc24, SPIN90 interacts with inactive Rab5/GDI2 and Gapex5, and this interaction finally facilitates Rab5 activation. In terms of Rab activation, the adaptor between Rab and its GEF has not been reported. A similar situation involving the Rabex5 and Rabaptin5 (the effector of Rabex5) complex has been reported;^22^ however, in this case, Rabex5 and Rabaptin5 exist as a complex in the cytosol, and the complex is recruited to the target membranes by immobilized Rab5. Moreover, Rabex5 and Rabaptin5 are tightly bound, and the complex does not dissociate, even after Rab5 activation^22^, whereas binding of SPIN90 and Gapex5 is signal-dependently regulated by the phosphorylation status of SPIN90, which is determined by EGF-induced ERK signaling. Collectively, the current study data demonstrate that SPIN90 physically links the GTPase Rab5 with its GEF, Gapex5, during EGF-mediated endocytosis, suggesting the possibility that SPIN90 serves as a signal-dependent, finely tuned regulator of Rab5 activation during EGFR endocytosis. Our findings also suggest the physiological importance of coupling EGF signaling with intracellular membrane trafficking.

## Supplementary information

Supplementary Figures and Legends

Supplementary Movie 1

## References

[1] Conner SD, Schmid SL (2003). Regulated portals of entry into the cell. Nature.

[2] Ivaska J, Heino J (2011). Cooperation between integrins and growth factor receptors in signaling and endocytosis. Annu Rev. Cell Dev. Biol..

[3] Barbieri MA (2000). Epidermal growth factor and membrane trafficking. EGF receptor activation of endocytosis requires Rab5a. J. Cell Biol..

[4] Stenmark H (2009). Rab GTPases as coordinators of vesicle traffic. Nat. Rev. Mol. Cell Biol..

[5] Zerial M, McBride H (2001). Rab proteins as membrane organizers. Nat. Rev. Mol. Cell Biol..

[6] Wu YW (2010). Membrane targeting mechanism of Rab GTPases elucidated by semisynthetic protein probes. Nat. Chem. Biol..

[7] Goud B, Salminen A, Walworth NC, Novick PJ (1988). A GTP-binding protein required for secretion rapidly associates with secretory vesicles and the plasma membrane in yeast. Cell.

[8] Pfeffer SR, Dirac-Svejstrup AB, Soldati T, Rab GDP (1995). dissociation inhibitor: putting rab GTPases in the right place. J. Biol. Chem..

[9] Shapiro AD, Pfeffer SR (1995). Quantitative analysis of the interactions between prenyl Rab9, GDP dissociation inhibitor-alpha, and guanine nucleotides. J. Biol. Chem..

[10] Sivars U, Aivazian D, Pfeffer SR (2003). Yip3 catalyses the dissociation of endosomal Rab-GDI complexes. Nature.

[11] Murata T (2006). The Legionella pneumophila effector protein DrrA is a Rab1 guanine nucleotide-exchange factor. Nat. Cell Biol..

[12] Machner MP, Isberg RR (2007). A bifunctional bacterial protein links GDI displacement to Rab1 activation. Science.

[13] Suh HY (2010). Structural insights into the dual nucleotide exchange and GDI displacement activity of SidM/DrrA. EMBO J..

[14] Rubino M, Miaczynska M, Lippe R, Zerial M (2000). Selective membrane recruitment of EEA1 suggests a role in directional transport of clathrin-coated vesicles to early endosomes. J. Biol. Chem..

[15] Christoforidis S, McBride HM, Burgoyne RD, Zerial M (1999). The Rab5 effector EEA1 is a core component of endosome docking. Nature.

[16] Rink J, Ghigo E, Kalaidzidis Y, Zerial M (2005). Rab conversion as a mechanism of progression from early to late endosomes. Cell.

[17] Nielsen E, Severin F, Backer JM, Hyman AA, Zerial M (1999). Rab5 regulates motility of early endosomes on microtubules. Nat. Cell Biol..

[18] Simonsen A (1998). EEA1 links PI(3)K function to Rab5 regulation of endosome fusion. Nature.

[19] Gorvel JP, Chavrier P, Zerial M, Gruenberg J (1991). rab5 controls early endosome fusion in vitro. Cell.

[20] Roberts RL, Barbieri MA, Ullrich J, Stahl PD (2000). Dynamics of rab5 activation in endocytosis and phagocytosis. J. Leukoc. Biol..

[21] Stenmark H (1994). Inhibition of rab5 GTPase activity stimulates membrane fusion in endocytosis. EMBO J..

[22] Horiuchi H (1997). A novel Rab5 GDP/GTP exchange factor complexed to Rabaptin-5 links nucleotide exchange to effector recruitment and function. Cell.

[23] Tall GG, Barbieri MA, Stahl PD, Horazdovsky BF (2001). Ras-activated endocytosis is mediated by the Rab5 guanine nucleotide exchange activity of RIN1. Dev. Cell.

[24] Topp JD, Gray NW, Gerard RD, Horazdovsky BF (2004). Alsin is a Rab5 and Rac1 guanine nucleotide exchange factor. J. Biol. Chem..

[25] Sato M (2005). Caenorhabditis elegans RME-6 is a novel regulator of RAB-5 at the clathrin-coated pit. Nat. Cell Biol..

[26] Delprato A, Merithew E, Lambright DG (2004). Structure, exchange determinants, and family-wide rab specificity of the tandem helical bundle and Vps9 domains of Rabex-5. Cell.

[27] Oh H (2013). SPIN90 knockdown attenuates the formation and movement of endosomal vesicles in the early stages of epidermal growth factor receptor endocytosis. PLoS ONE.

[28] Kim SH (2006). Interaction of SPIN90 with syndapin is implicated in clathrin-mediated endocytic pathway in fibroblasts. Genes Cells.

[29] Simpson F (1999). SH3-domain-containing proteins function at distinct steps in clathrin-coated vesicle formation. Nat. Cell Biol..

[30] Kim Y (2005). Interaction of SPIN90 with dynamin I and its participation in synaptic vesicle endocytosis. J. Neurosci..

[31] Blumer J (2013). RabGEFs are a major determinant for specific Rab membrane targeting. J. Cell Biol..

[32] Miaczynska M (2004). APPL proteins link Rab5 to nuclear signal transduction via an endosomal compartment. Cell.

[33] Bucci C (1992). The small GTPase rab5 functions as a regulatory factor in the early endocytic pathway. Cell.

[34] Lachmann J, Ungermann C, Engelbrecht-Vandre S (2011). Rab GTPases and tethering in the yeast endocytic pathway. Small GTPases.

[35] Butty AC (2002). A positive feedback loop stabilizes the guanine-nucleotide exchange factor Cdc24 at sites of polarization. EMBO J..

