# Supplementary material for: SPIN90, an adaptor protein, alters the proximity between Rab5 and Gapex5 and facilitates Rab5 activation during EGF endocytosis
Source: Exp Mol Med. 2019 Jul 29;51(7):85. doi: 10.1038/s12276-019-0284-5 (PMC6802610; doi:10.1038/s12276-019-0284-5)
Supplement: Supplementary file 1 — Supplementary Figures and Legends [file 12276_2019_284_MOESM1_ESM.docx]

Supplementary Information

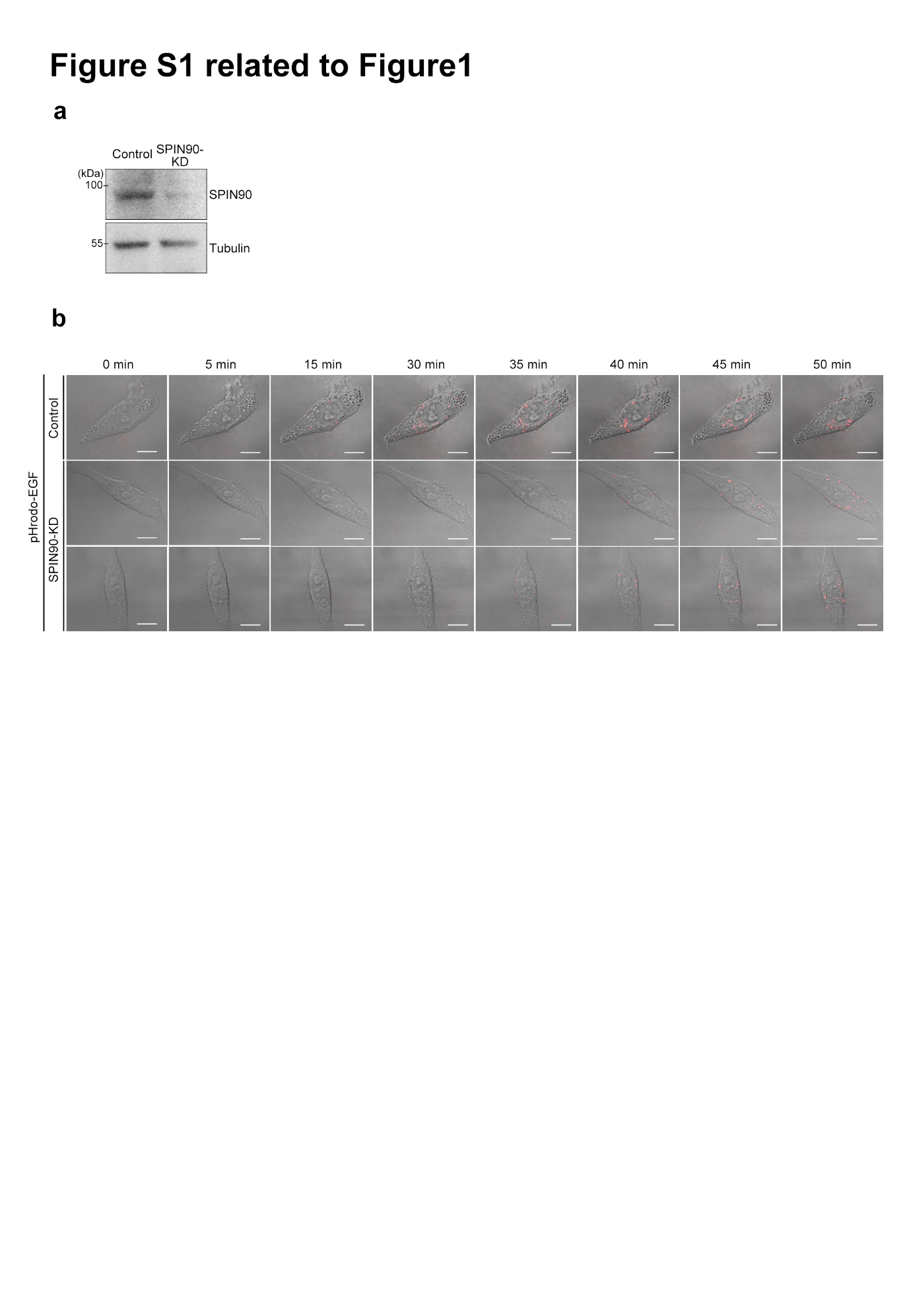


Fig. S1. SPIN90-KD cells exhibit delayed vesicle trafficking during EGF-mediated endocytosis. (a) shRNA-induced knockdown of SPIN90 in HeLa cells was verified by Western blotting with an anti-SPIN90 antibody. (b) SPIN90-KD and control HeLa cells were treated with 40 ng/ml pHrodo EGF, and fluorescence signals (red) were monitored by confocal microscopy. Time-lapse images were acquired for 50 min, and representative images from the indicated time points are shown (see Movie S1). Scale bars, 10 μm.

**
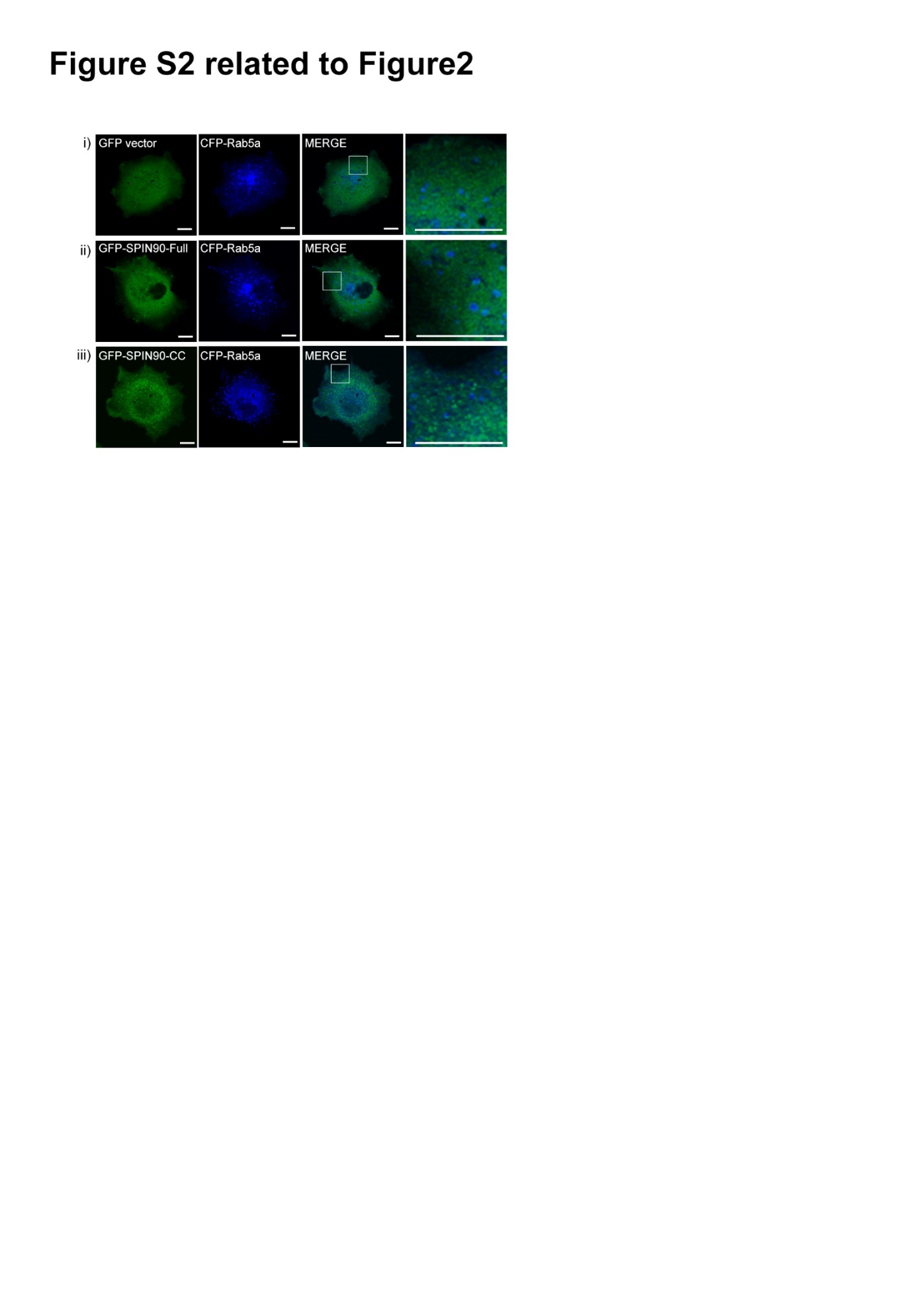
**

Fig. S2. Cellular localizations of SPIN90 and Rab5a. COS-7 cells transiently co-transfected with vectors encoding CFP-tagged Rab5 and GFP-tagged SPIN90 variants (WT or -CC) were serum-starved for 16 h, fixed and observed by confocal microscopy. Scale bars, 10 μm.


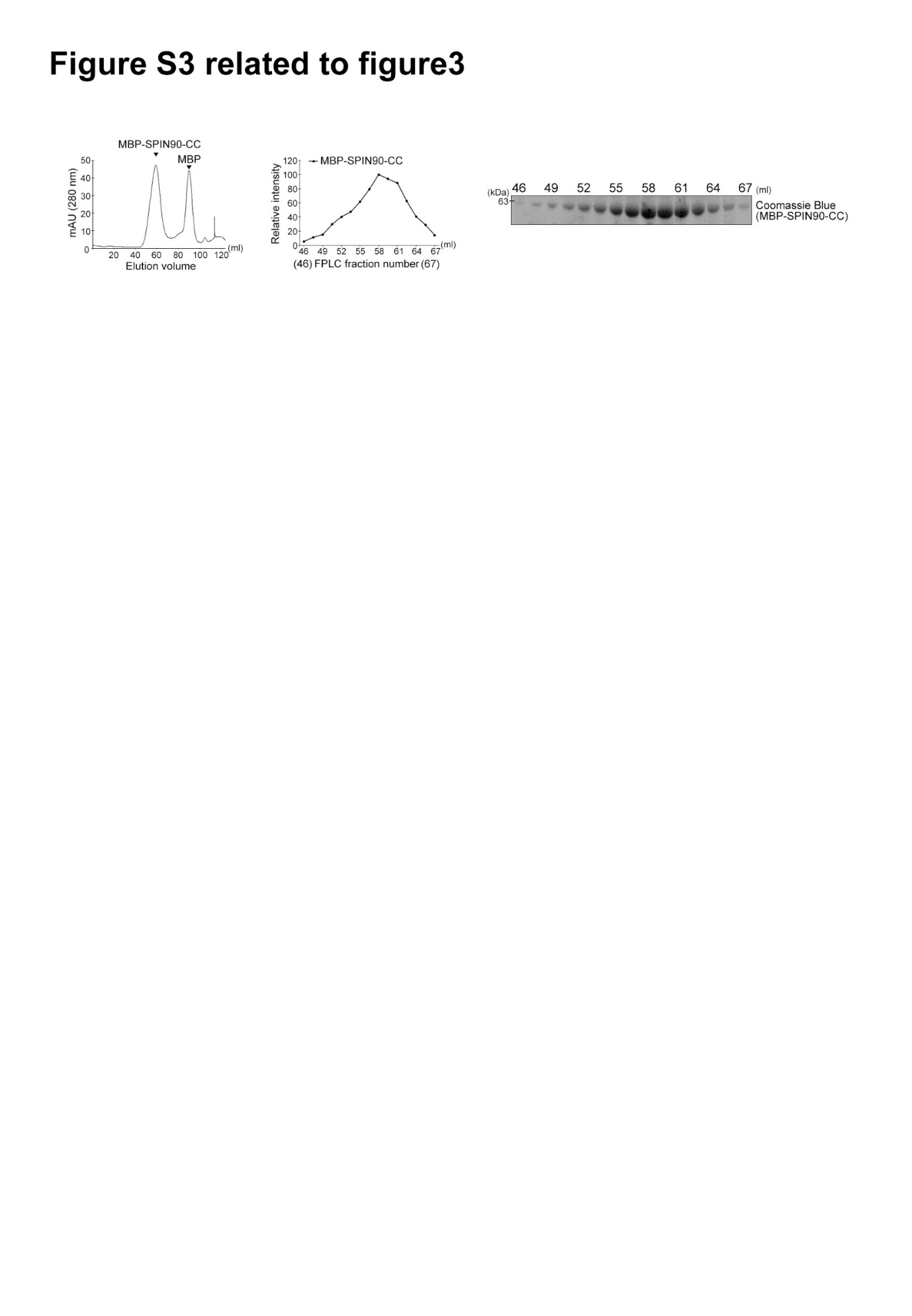
Fig. S3. MBP-SPIN90-CC domains are highly aggregated. Purified MBP-SPIN90-CC domains were analyzed by size-exclusion gel-filtration chromatography.


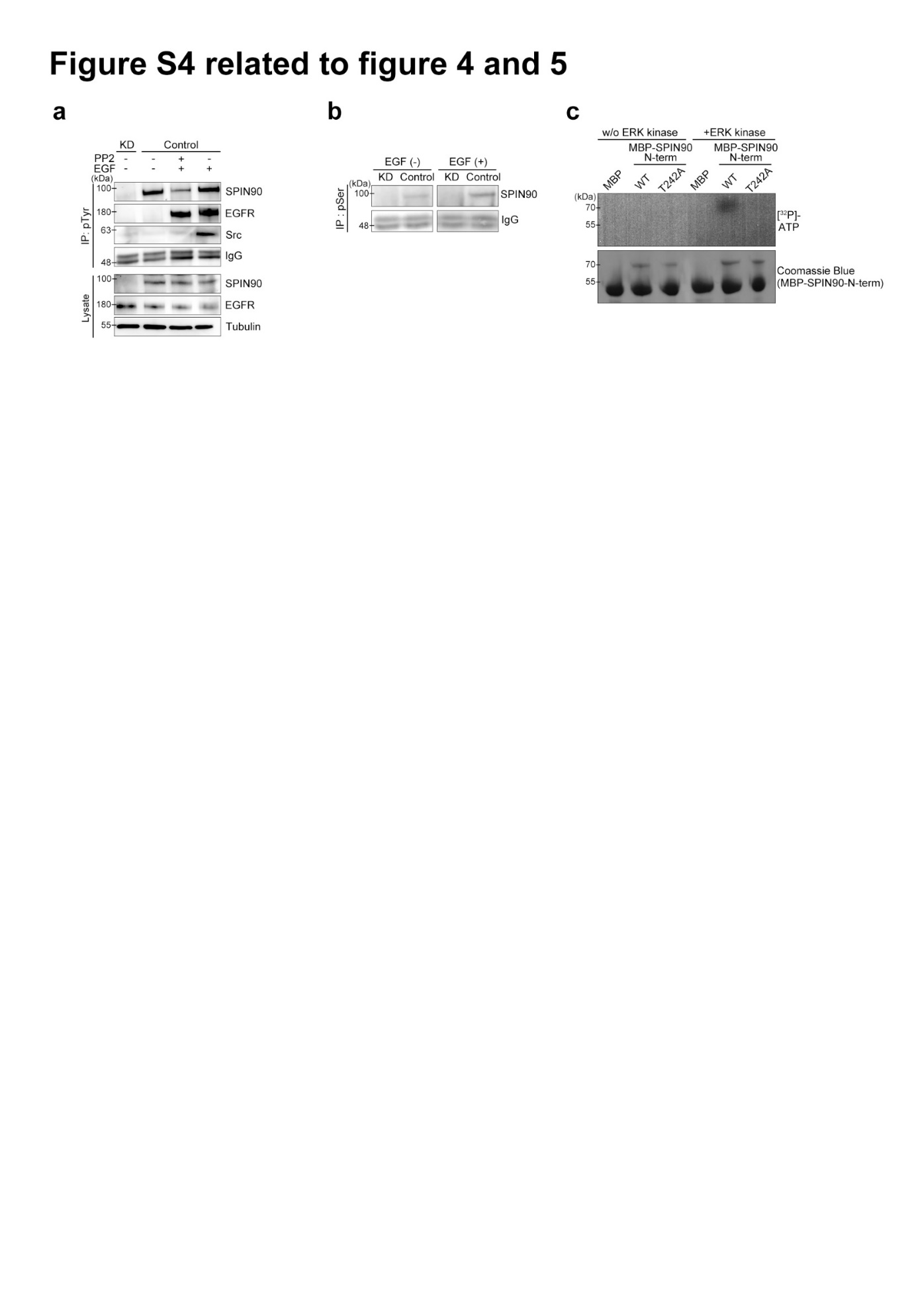
Fig. S4. EGF-induced interaction of SPIN90 with Gapex5 is dependent on threonine phosphorylation of SPIN90. (a) HEK293T cells were treated with 40 ng/ml EGF in the absence or presence of the selective Src family inhibitor, PP2 (1 μg/ml), lysed, and immunoprecipitated with an anti-pTyr antibody. (b) SPIN90-KD and control HeLa cells treated with EGF were immunoprecipitated with an anti-pSer antibody and analyzed by Western blotting. (c) HEK293T cells transfected with MBP-tagged SPIN90 N-term variants (WT or T242A) were used for *in vitro* ERK kinase assays with [^32^P]-ATP.


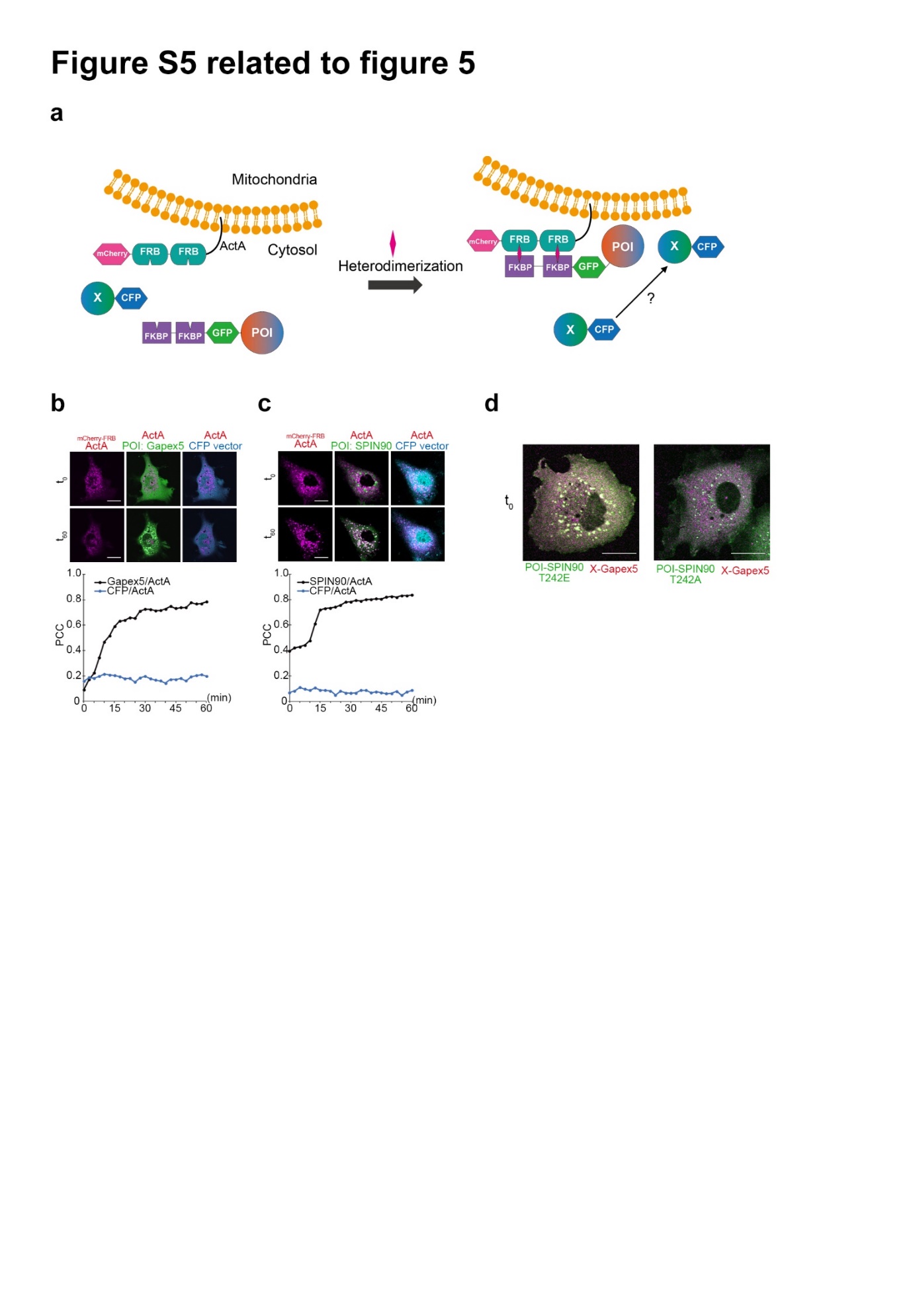
Fig. S5. Artificial targeting of Gapex5 to mitochondrial membranes. (a) Schematic diagram of the rapamycin-induced protein heterodimerization system and domain structure of fluorescent fusion proteins. (b and c) Cos-7 cells were transfected with mitochondrial-localized mCherry-FRB ActA protein, CFP-empty vector (control for CFP-tagged X proteins) and FKBP-GFP-Gapex5 (b) or FKBP-GFP-SPIN90 (c). Time-lapse images were acquired every 20 s for 1 h after the addition of heterodimerizer; initial (t_0_) and final (t_60_) images are shown. The Pearson’s correlation coefficient (PCC) for the colocalization between mCherry-FRB ActA and CFP-empty vector, between mCherry-FRB ActA and FKBP-GFP-Gapex5 (b) or between mCherry-FRB ActA and FKBP-GFP-SPIN90 (c) were calculated. Scale bars, 20 μm. (d) Initial (t_0_) merged images corresponding Fig 5F (POI, SPIN90-T242E and X, Gapex5) and 5G (POI, SPIN90-T242A and X, Gapex5). Scale bar, 20 μm.

Legend for Supplemental Movie S1
SPIN90-KD and control HeLa cells were treated with 40 ng/ml pHrodo EGF, and fluorescence signals (red) were monitored by confocal microscopy. Time-lapse images were acquired every 10 s.
